# A single centre retrospective study of systemic reactions to subcutaneous immunotherapy

**DOI:** 10.1186/s13223-020-00491-5

**Published:** 2020-11-02

**Authors:** Kara Robertson, Nazanin Montazeri, Urvashi Shelke, Samira Jeimy, Harold Kim

**Affiliations:** 1 Clinical Immunology and Allergy, Department of Medicine, St. Joseph’s Hospital, Western University, 268 Grosvenor Street, London, ON N6A 4V2 Canada; 2 Department of Sociology and Legal Studies, Faculty of Arts, University of Waterloo, 200 University Avenue West, Waterloo, ON N2L 3G1 Canada; 3 Division of Clinical Immunology & Allergy, Department of Medicine, McMaster University, Belgage Medical Arts Centre Suite 205, 525 Belmont Avenue West, Kitchener, ON N2M 5E2 Canada

**Keywords:** Subcutaneous immunotherapy, SCIT, Anaphylaxis, Systemic reaction, Allergic rhinitis, Aeroallergen, Epinephrine

## Abstract

**Background:**

Subcutaneous immunotherapy (SCIT) is the standard approach for treating patients with sensitizations to aeroallergens. However, immunotherapy can trigger severe systemic reactions if delivered inappropriately or to high risk patients. We sought to characterize and quantify SCIT systemic reactions requiring epinephrine administration during a 6-year period in a Canadian setting following the recommendations for components and dosages published in the 2010 Canadian Society of Allergy and Clinical Immunology (CSACI) Immunotherapy Manual.

**Methods:**

A single centre retrospective chart review was performed for all patients with systemic reactions to subcutaneous immunotherapy requiring intramuscular epinephrine injection between January 2011 and October 2017. Each systemic reaction requiring epinephrine was reviewed for baseline patient characteristics, details of the reaction, and reaction severity. Research ethics approval was obtained through McMaster University.

**Results:**

28 of 380 patients experienced a systemic reaction requiring epinephrine administration, with an incidence rate of 1 per 1,047 injection visits (0.095%). 26 of the 28 reactions occurred within the mandatory 30-minute observation period post allergen immunotherapy. Of the 28 patients that experienced a systemic reaction to SCIT, 11 patients had asthma and 5 patients had a history of possible food allergy. All of the systemic reactions occurred during injections from vial number 4, and five patients reacted to their first shot of a re-ordered extract. 10 of the 28 patients required more than one intramuscular injection of epinephrine, and 20 of 28 patients were transferred to the hospital by ambulance.

**Conclusions:**

This is the first Canadian study to review patients with systemic reactions to subcutaneous immunotherapy. Several best practice methods were employed throughout the study to optimize subcutaneous delivery of immunotherapy extract, and our recorded per injection incidence rate for systemic reactions was comparable or below the rate published in similar studies. The recommendations in the CSACI Immunotherapy Manual provide an approach to standardizing prescriptions for SCIT to maximize immunotherapy efficacy and reduce the risk of systemic reactions, though similar studies in larger multicenter settings are needed to confirm these observations. These observations provide important objective information to clinicians about the potential risks for systemic reactions in patients considering SCIT.

## Background

Subcutaneous immunotherapy (SCIT) is the standard approach for treating patients with sensitizations to aeroallergens if medical therapy is ineffective or not preferred [1], with a strong evidence base supporting its use [1, 2]. Systemic reactions represent the major adverse effect of immunotherapy. Risk factors for systemic reactions include symptomatic asthma, injections from new vials, injections during symptomatic seasons, dosing errors, history of previous systemic reactions and the use of beta-blockers [2]. These reactions can be severe, and in rare cases, fatal [1].

Multiple studies have tried to characterize the underlying risks and quantify the incidence of systemic reactions and deaths due to SCIT. Several surveys have reported that the fatality rate is estimated to be approximately 1 in 2.5 million injections. [3–5] Although these fatal reactions are rare, the generally accepted incidence of systemic reactions per injection is between 0.1% and 0.3% [6–8] Furthermore, retrospective and prospective studies of inhalant immunotherapy following conventional schedules have been reported from the United States [7–9] Turkey [10, 11], Israel [12], Portugal [13], Italy [14, 15], and Spain [16]. However, Canadian data has not yet been published.

To standardize the delivery of SCIT, recommendations for immunotherapy extract components and dosages were published in the 2010 Canadian Society of Allergy and Clinical Immunology (CSACI) Immunotherapy Manual. Appropriate allergen dosing, mixing and build up schedules are provided in order to improve the efficacy and safety of SCIT. The objectives of this study were to characterize and quantify SCIT systemic reactions requiring epinephrine administration in a Canadian setting from 2011 to 2017 following the publication of the 2010 CSACI Immunotherapy Manual [17].

## Methods

A retrospective chart review was performed for all patients with systemic reactions to aeroallergen subcutaneous immunotherapy requiring intramuscular epinephrine injection at a single referral clinic of an Allergist in Kitchener, Ontario, Canada. The data was collected for reactions to the subcutaneous injection of glycerinated extracts (Hollister Stier, Spokane, Washington, USA) requiring epinephrine that occurred between January 2011 and October 2017, following the publication and adoption of the recommendations within the 2010 CSACI Immunotherapy Manual. All patients with a clinical history of asthma were first assessed with spirometry prior to initiation of SCIT to ensure an FEV1 greater than 80%. Asthma symptoms were well controlled in all patients prior to each injection. Any first injections of new extract were administered with a volume reduction of at least 50%. The total number of injections administered at the clinic during the study period was also documented.

Each systemic reaction requiring epinephrine was reviewed for baseline patient characteristics (age, sex, past medical history including the presence of asthma and food allergies, use of beta-blockers, angiotensin converting enzyme inhibitors or angiotensin receptor blockers, and positive skin tests prior to starting SCIT), and the details of the reaction (severity, date and season in which the reaction occurred, the dose of immunotherapy and whether the extract was a new reorder, the allergens in the extract, time course, specific symptoms, blood pressure, treatments including the epinephrine dose and subsequent doses, and transfer to the hospital).

Reaction severity was determined by the 2010 World Allergy Organization (WAO) subcutaneous immunotherapy systemic reaction grading system (Table 1) [6].

**Table 1:** 2010 World Allergy Organization (WAO) subcutaneous immunotherapy systemic reaction grading system

WAO systemic reaction grade	Reaction characteristics
1	Symptoms from one organ system, excluding asthma, gastrointestinal symptoms or cardiovascular symptoms
2	Asthma, gastrointestinal symptoms or cardiovascular symptoms
3	Patients with symptoms from multiple organ systems, or throat tightness/discomfort suggestive of upper airway edema
4	Hypotension (systolic blood pressure of less than 90 mmHg or less than 30% of baseline) or respiratory failure
5	Death

*mmHg* millimeter of mercury

Research ethics approval was obtained through McMaster University in Hamilton, Ontario, Canada (Project number: 4213-C).

## Results

Between January 2011 and October 2017, 380 patients received aeroallergen SCIT with a total of 29,334 injections administered. Of these 380 patients, 28 patients (7.4%) experienced systemic reactions requiring epinephrine administration (Table 2). The incidence rate of reactions requiring epinephrine was 1 per 1,047 injection visits (0.095%).

**Table 2:** Patient demographic and clinical characteristics

Characteristic	Patients with systemic reaction
Median age—yr	34
Age range—yr	8–60
Male patients, n (%)	19 (68.9)
Angiotensin converting enzyme inhibitor or angiotensin receptor blocker use, n (%)	0 (0)
Beta-blocker use, n (%)	0 (0)
Asthma, n (%)	11 (39.3)
Grade 1 reaction, n (%)	8 (28.6)
Grade 2 reaction, n (%)	9 (32.1)
Grade 3 reaction, n (%)	8 (28.6)
Grade 4 reaction, n (%)	3 (10.7)

In this population of patients that experienced a systemic reaction to SCIT, no patients were on beta-blockers, angiotensin converting enzyme inhibitors or angiotensin receptor blockers. However, there was a male predominance in the 28 patients that experienced a systemic reaction, with 19 (68.9%) males and 9 (32.1%) females. No age predilection could be identified, with an age range between 8 to 60 years (median 34 years; four patients under the age of 18). The number of positive skin tests to environmental allergens prior to starting SCIT ranged from 3 to 11.

Of the 28 patients that experienced a systemic reaction to SCIT, 11 patients had asthma (39.3%) and 5 patients (17.9%) had a history of possible food allergy. Of these five, 4 had confirmed food allergy, with allergies to crustaceans, peanut, tree nuts and carrots.

There was no statistically significant seasonal predominance for the timing of systemic reactions; however, there was an increase in the number of reactions during the winter months. Reactions occurred during all seasons with 5 reactions (17.9%) in the spring, 5 in the summer (17.9%), 8 in the autumn (28.6%), and 10 in the winter (35.7%).

The injected extracts contained different combinations of dust mite, grass, ragweed, trees, mold, cat, and dog allergens. The number of allergen extracts administered on one visit with one or two injections ranged from 2 to 7, with different tree species considered a single aggregate allergen. All systemic reactions occurred during injections from vial number 4 (most concentrated extract), with injection volumes ranging from 0.05 ml to 0.5 ml. Five (17.9%) patients reacted to their first injection of a re-ordered (*i.e.* new vial of) extract.

Out of the 28 reactions identified, 26 reactions (92.9%) occurred within the mandatory 30-min observation period post allergen immunotherapy as recommended in the 2010 CSACI Immunotherapy Manual. Only 2 reactions (7.1%) occurred outside of the mandatory observation period, with one patient experiencing a reaction shortly after departing the observation environment, and another reaction occurring approximately 90 min after the injection was given.

There was an even distribution of the severity of reactions. Eight patients (28.6%) experienced grade 1 reactions, 9 patients (32.1%) experienced grade 2 reactions, 8 patients (28.6%) experienced grade 3 reactions, and 3 patients (10.7%) experienced grade 4 reactions (Table 3) according to the WAO grading scale [6]. No grade 5 reactions occurred.

**Table 3:** Summary of patients with grade 4 reactions

Age—yr, gender	Reaction year, season	Asthma	Food allergies	SCIT dose	New serum	SCIT content	Reaction onset and symptom description	Treatment
53, male	2012, winter	Yes	None	Vial 4, 0.25 ml	No	Grass, Ragweed, Trees	Few minutes; flushing, throat tightness, feeling unwell, systolic blood pressure 70 mmHg	Epinephrine 0.5 ml IM then 0.3 ml IM, salbutamol and normal saline. Patient was transferred to the hospital.
16, male	2013, autumn	Yes	Peanut and tree nuts	Vial 4, 0.25 ml	Yes	Dust mites, Ragweed, Trees, Mold, Cat	30 min; shortness of breath, urticaria, flushing, throat tightness, nausea, swelling of the eyes, chest tightness, blood pressure 49/29 upon standing in the hospital	Epinephrine 0.5 ml IM in clinic followed by two injections of epinephrine 0.3 ml IM in the hospital, salbutamol, prednisone, antihistamines and normal saline. Patient was transferred to the hospital
35, male	2016, winter	No	None	Vial 4, 0.10 ml	Yes	Dust mites, Grass, Ragweed, Trees, Cat	Immediate; flushing, feeling unwell, lightheadedness, blood pressure 65/40 mmHg after epinephrine was given	Epinephrine 0.5 ml IM then 0.3 ml IM and normal saline. Patient was transferred to the hospital

*SCIT* subcutaneous immunotherapy, *mmHg* millimeter of mercury, *IM* intramuscular

Of the 28 patients receiving epinephrine, 10 patients (35.7%) received more than one intramuscular injection of epinephrine, and 1 patient (3.6%) received a dose of epinephrine subcutaneously (0.1 ml of 1:1000 epinephrine) at the SCIT injection site in addition to the initial dose of intramuscular epinephrine. In total, 20 (71.4%) patients were transferred to the hospital by ambulance.

## Discussion

This is the first Canadian study to review patients with systemic reactions to aeroallergen subcutaneous immunotherapy. The 2010 CSACI Immunotherapy Manual [17] provided recommendations for SCIT administration, including standardized dosages (when available) and immunotherapy extract components based on published data. In our study, we focused on documenting the patient characteristics and reaction details following the adoption of these guidelines in an attempt to elicit common predisposing risk factors for systemic reactions while on subcutaneous immunotherapy. Our recorded incidence rate for systemic reactions was approximately 0.095% per injection, comparable or below the rate published in similar studies (0.1% to 0.3%) [7–9, 18]. Our low incidence rate may simply have been skewed by the inclusion criteria used in our retrospective review (administration of intramuscular epinephrine). However, we felt this requirement would be the most definitive method of confirming a true reaction. Therefore, patients with mild systemic reactions who did not receive epinephrine were excluded from analysis.

Several precautionary methods were employed throughout the study to optimize subcutaneous delivery of immunotherapy extract. Before starting SCIT or during SCIT, most patients (26/28) underwent an ultrasonographic assessment of the posterolateral arm to determine the subcutaneous soft tissue depth to decrease the probability of an intramuscular delivery. Furthermore, extract was injected at a 45-degree angle with cautious needle advancement to increase the probability of subcutaneous extract delivery. These approaches to injections may have contributed to a lower observed risk of reaction per injection.

The risk factors for systemic reactions are well documented [2] and our study supports these findings. In our study, 39.3% of the patients requiring epinephrine had asthma, although injections are generally withheld when patients have symptoms of uncontrolled asthma. The presence of asthma alone as an independent risk factor further reinforces the hypothesis that patients with asthma are at a higher risk for systemic reactions, and SCIT should be administered with great caution.

Five (17.9%) of the patients requiring epinephrine reacted to the first injection from a newly reordered extract. Furthermore, 2 of the 3 patients who experienced grade 4 reactions had also received their dose from a newly reordered extract. These observations support the hypothesis that the significant differences in concentration between that of an expiring extract and a newly reordered extract are sufficient to evoke severe systemic reactions. Therefore, the dose for the first injection from a new reordered extract should be reduced to 50% or less than the previous maintenance dose to reduce the probability of evoking a systemic reaction.

Within our data, we observed more reactions during the winter season (35.7%) than any other season. Although reactions occurred year-round, the increased frequency of reactions during the winter may in turn reflect the increased volume of immunotherapy initiations during the autumn season. Consequently, these new initiations would then be receiving injections from vial number 4 during the winter season, which was associated with the highest risk of systemic reaction.

Amongst the most severe reactions, we observed that these patients were between the ages of 16 to 53 years. All three patients were being treated for multiple allergies, and the reaction onset was within 30 min of the injection. Multiple risk factors for an increased probability of reaction and reaction severity were identified, with asthma in the past history of a majority of these patients, and a majority of these reactions occurring during winter. Furthermore, a majority of patients were receiving the first injection from a newly reordered vial. These patients all required multiple doses of intramuscular epinephrine and were transferred to the hospital for further management and observation.

There are some limitations to this retrospective study. Although a prospective study would provide more consistent data and be powered for generalizability of predictive factors, we do not believe that a prospective study would change the likelihood of epinephrine administration. This study was also limited to patients on SCIT for aeroallergens and did not consider the incidence or risk factors for patients on venom immunotherapy, limiting the generalizability of these findings to other SCIT populations. Furthermore, the narrow geographic and demographic patient profile from this single-center study may limit the generalizability of our results. Similar studies in larger multicenter settings are needed to confirm these observations.

## Conclusion

Our findings complement the available data on systemic reactions with subcutaneous immunotherapy and provide further information about the rate and nature of reactions requiring epinephrine [19]. The recommendations in the CSACI Immunotherapy Manual provide an approach to standardizing prescriptions for SCIT to maximize immunotherapy efficacy and reduce the risk of systemic reactions. Taken together, these observations provide important objective information to clinicians about the potential risks for systemic reactions in patients considering SCIT.

## Data Availability

The datasets used and analyzed during the current study are available from the corresponding author upon reasonable request.

## Abbreviations

SCIT: Subcutaneous immunotherapy
CSACI: Canadian Society of Allergy and Clinical Immunology
WAO: World Allergy Organization
Ml: Milliliter
MmHg: Millimeter of mercury
IM: Intramuscular
